# Gene Expression Data from the Moon Jelly, *Aurelia*, Provide Insights into the Evolution of the Combinatorial Code Controlling Animal Sense Organ Development

**DOI:** 10.1371/journal.pone.0132544

**Published:** 2015-07-30

**Authors:** Nagayasu Nakanishi, Anthony C. Camara, David C. Yuan, David A. Gold, David K. Jacobs

**Affiliations:** Department of Ecology and Evolutionary Biology, UCLA, Los Angeles, California, United States of America; Sars International Centre for Marine Molecular Biology, NORWAY

## Abstract

In Bilateria, Pax6, Six, Eya and Dach families of transcription factors underlie the development and evolution of morphologically and phyletically distinct eyes, including the compound eyes in *Drosophila* and the camera-type eyes in vertebrates, indicating that bilaterian eyes evolved under the strong influence of ancestral developmental gene regulation. However the conservation in eye developmental genetics deeper in the Eumetazoa, and the origin of the conserved gene regulatory apparatus controlling eye development remain unclear due to limited comparative developmental data from Cnidaria. Here we show in the eye-bearing scyphozoan cnidarian *Aurelia* that the ectodermal photosensory domain of the developing medusa sensory structure known as the rhopalium expresses *sine oculis* (*so*)*/six1/2* and *eyes absent/eya*, but not *optix/six3/6* or *pax (A&B)*. In addition, the *so* and *eya* co-expression domain encompasses the region of active cell proliferation, neurogenesis, and mechanoreceptor development in rhopalia. Consistent with the role of *so* and *eya* in rhopalial development, developmental transcriptome data across *Aurelia* life cycle stages show upregulation of *so* and *eya*, but not *optix* or *pax (A&B)*, during medusa formation. Moreover, *pax6* and *dach* are absent in the *Aurelia* genome, and thus are not required for eye development in *Aurelia*. Our data are consistent with *so* and *eya*, but not *optix*, *pax* or *dach*, having conserved functions in sensory structure specification across Eumetazoa. The lability of developmental components including Pax genes relative to *so*-*eya* is consistent with a model of sense organ development and evolution that involved the lineage specific modification of a combinatorial code that specifies animal sense organs.

## Introduction

Eye evolution has fascinated biologists since Darwin. He [1] considered an eye one of “the organs of extreme perfection and complication” that appeared “absurd in the highest possible degree” to have resulted from natural selection. Nevertheless he presented evidence—gradations in the degree of morphological complexity in crustacean photoreceptors—that natural selection would indeed be a sufficient evolutionary mechanism for the generation of eyes. Almost a century later, Salvini-Plawen and Mayr [2] extended Darwin’s analysis by examining morphologies of eyes across Metazoa at the electron microscopic level, and concluded that eyes evolved at least 40 times independently by means of natural selection. However, developmental genetics provided a surprise; vertebrate eyes and insect eyes, previously thought to have arisen independently, develop via a conserved gene regulatory network, the so-called “retinal determination gene network (RDGN)”, consisting of homologous transcription factors, *Pax6*, *Six*, *Eya* and *Dach* (named *twin of eyeless/eyeless*, *sine oculis* (*so*)*/optix*, *eyes absent* and *dachshund* (*dac*), respectively, in insects; reviewed in [3]). This suggested deep homology of eye development across the Bilateria. Yet none of these transcription factors, singly or in combination, function exclusively in eye development; they are also involved in the development of other tissue structures, such as ears, muscles and kidneys, potentially suggesting more basal functions earlier in animal evolution as well as a common role in sense organ development [4]. Here we explore the role of this suite of developmental genes in *Aurelia* eye development as a step in generating a more complete understanding of the role of these genes in eye development in the Cnidaria, the sister taxon to the well-studied Bilatera [5, 6].

**Fig 1:**
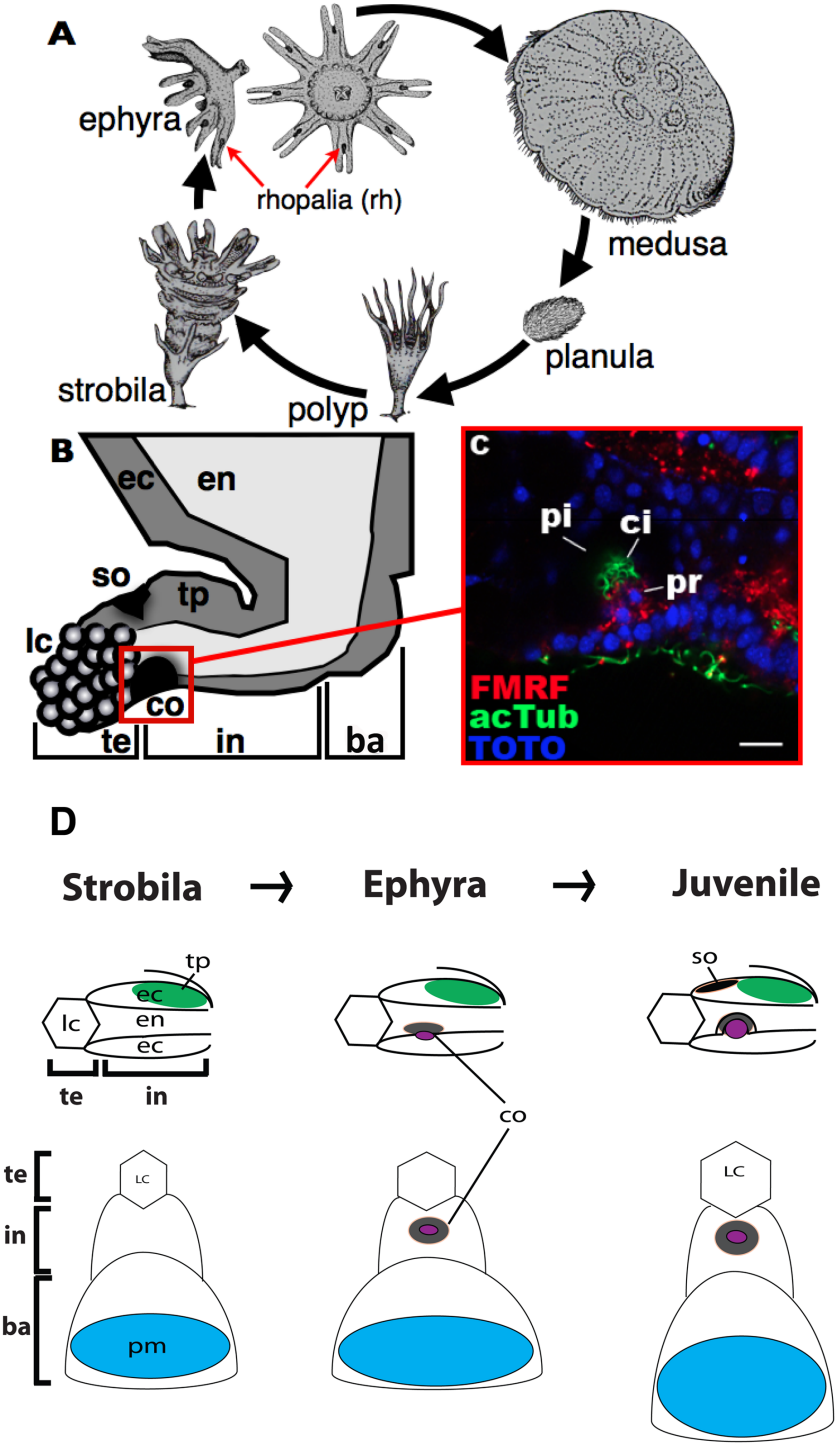
Scyphozoan life cycle and morphology of a scyphozoan rhopalium. A: Life cycle stages of the scyphozoan *Aurelia*. Scyphozoan ephyrae develop through transverse fission of polyps, a process known as strobilation (the “strobila” stage). Rhopalia (rh) begin development at the margin of each segment during strobilation. B: A schematic lateral view of the rhopalium in a juvenile medusa (“metephyra”). The distal end is to the left, the proximal end to the right. Oral is down, aboral up. Each rhopalium consists of terminal (te), intermediate (in) and basal (ba) regions, and has a gravity-sensitive organ consisting of the lithocyst (lc) and the touch plate (tp), a pigment-cup ocellus (co), and a pigment-spot ocellus (so). The boxed region is the cup-ocellus (co) shown in C. Morphological and behavioral evidence supports the photosensory function of the cup ocelli, but not of the spot ocelli [10–12], and thus this study focuses on analyzing gene expression in relation to the development of the former, but not the latter, structure. C: A confocal section of the cup-ocellus at the metephyra stage labeled with antibodies against FMRFamide-like neuropeptides (FMRF) and acetylated ∂-tubulin (acTub). Note basal cilia (ci) of photoreceptor cells (pr). The dark region surrounding the photoreceptor cells (pr) is the endodermal pigment layer (pi). Nuclei are labeled with the fluorescent dye TOTO. D: Schematics of developing rhopalia from late strobila (“Strobila”), through ephyra, into metephyra (“Juvenile”) stages. The top row shows the lateral view of the terminal (te) and intermediate (in) regions of a developing rhopalium; the distal end is to the left, the oral side down. The bottom row shows the oral view of a developing rhopalium; the distal end is to the top, the proximal end to the bottom. In the developing cup ocellus (co), the domain of ciliated ectodermal photoreceptor cells is shown in purple, while the domain of endodermal pigment cells is shown in grey. At strobilation, the gravity-sensitive organ—the lithocyst (lc) and the touch plate (tp)—begin development first, followed by the pacemaker neurons (pm) located in the proximal-most, basal region (ba) of the rhopalial ectoderm. Cup ocelli (co) start to differentiate in free-swimming ephyrae, while the pigment-spot ocelli (so) develop later at the metephyra stage. Note that the developing photosensory tissue of the cup ocellus is located in the oral-medial ectoderm of the intermediate region of the rhopalium. Abbreviations: en endoderm; ca rhopalar canal. Scale bars: 100 μm (A), 10 μm (B).

In the Cnidaria, Medusozoa (i.e. jellyfishes) have diverse photoreceptor types ranging from simple eye-spots to complex eyes with retinas (see [7] for a review). The medusozoan life history usually consists of a motile planula stage, a sessile polyp stage and a free-swimming, sexual medusa stage (e.g. Fig 1A). Distinct ocular structures are typically found at the bell margin of the medusa (but see [8, 9] for description of putative eye-like structures in a planula larva and a polyp, respectively), either at the base of the tentacles (in Hydrozoa), or in the club-shaped sensory structures called the rhopalium that usually occurs in multiples of four (at least eight in Scyphozoa, Fig 1A; four in Cubozoa).

Cnidarians have some but not all of the components of the RDGN in their genomes. Members of the paired box containing *Pax* gene family named *paxA*, *B*, and *C/D*, (orthologous to bilaterian *pox neuro*, *pax2/5/8* and *pax3/7*, respectively) are present, but *pax6* orthologs are lacking [13]. Orthologs to all three members of the *Six* gene family (*six1/2/so*, *six3/6/optix* and *six4/5/myotonix*) [14], as well as an *eya* ortholog [15] are present. Paralleling the bilaterian condition, the *Six* genes (*so* and *optix*) and *eya* are expressed in the eyes of the adult hydrozoan jellyfish *Cladonema radiatum* [14, 15], and *so* is known to be upregulated in the rhopalia of the scyphozoan *Aurelia* in developing medusae [16]. In addition, *so* and *optix* are expressed in regenerating eyes in *Cladonema*, consistent with their function in eye regeneration [14]. In contrast, cnidarian *pax* gene functions appear divergent relative to the Bilateria; *paxA*, but not *paxB*, is expressed in the *Cladonema* eye, while *paxB* is expressed in the retina of the highly sophisticated lensed-eye of the cubozoan *Tripedalia cystophora* in adult [17]. In all these cases, however, gene expression patterns during, or preceding, normal eye development have not been examined, and thus whether these genes have roles in normal eye development in cnidarians is unclear.

Data from the Scyphozoa, an additional eye-bearing medusozoan lineage, are more limited than such data from Hydrozoa [16], and could help clarify the apparent evolutionary persistence of *six* and *eya* function in cnidarian eye evolution, as well as the extent to which *pax* gene functions—as inferred from their expression domains—have been modified in cnidarian evolution. In addition, the pattern of scyphozoan eye development—a framework needed to interpret developmental gene expression patterns—has been previously documented (see below; [18]). Thus, comparative data from this additional early-diverging cnidarian lineage are important for our understanding of the evolution of the RDGN within Cnidaria, as well as the state of this gene regulatory network basal to the Bilateria.

The scyphozoan rhopalium consists of discrete terminal, intermediate and basal regions along the distal-proximal axis, which are subdivided by two shallow grooves (Fig 1B; [18, 19]). In *Aurelia*, each rhopalium has two types of presumptive photoreceptors, cup ocelli and spot ocelli located in the intermediate segment on the oral side and the aboral side, respectively (Fig 1B). The cup-shaped layer of endodermal pigment cells of the cup ocellus contains basal ectodermal photosensory cells, with basal-facing coiled cilia oriented toward the endodermal pigment cup (Fig 1C; [18, 20]). The spot ocellus consists of a patch of ectodermal pigment cells with long cilia that have knobbed tips [18, 20, 21]. Morphological and behavioral evidence strongly supports a photosensitive function of the cup ocellus, but not of the spot ocellus [10–12]. Thus we here focus on analyzing gene expression during the development of the cup ocellus, and refer to the cup ocellus as an eye, based on the presence of photoreceptor cells backed by pigment cells [22]. In addition to ocelli, each rhopalium develops a mechanoreceptor consisting of a distally located lithocyst (containing gypsum crystals acting as a localized mass; lc in Fig 1B) in the terminal segment and an aboral mechanosensory epithelium or "touch plate" in the intermediate segment (tp in Fig 1B). The lithocyst and touch plate are components of an ear-like inertial/gravitational sensory organ. Photo- and mechano-sensory stimuli perceived at rhopalia are thought to be communicated through the rhopalial nervous system to the pacemaker neurons located in the basal segment of each rhopalium; the pacemaker neurons then effect stimulus-dependent locomotory responses (e.g. changes in pulsation rate upon illumination) via modulation of electrical input into the motor nerve net (“MNN”) that innervates swimming muscles of the bell [18, 23–26].

Rhopalia begin development during medusa formation. In Scyphozoa, medusa formation occurs by strobilation, in which a polyp undergoes transverse fission to give rise to one or more free-swimming early medusae referred to as ephyrae (at the “strobila” stage; Fig 1A). During strobilation, development proceeds sequentially from the oral pole to the aboral pole, and thus upper segments are more developed than the lower segments. The bottom portion of the strobila transforms into a polyp. In developing rhopalia during strobilation, the gravity-sensitive organ (the lithocyst and the touch plate) begins development first (Fig 1D), followed by the pacemaker neurons [18]. Ocelli do not begin development until the release of ephyrae [18]. In the newly-released ephyrae, subepidermal ciliated photoreceptor cells of the cup-ocelli and associated pigment cells start to differentiate from ectoderm and endoderm, respectively (Fig 1D). At a juvenile “metephyra” stage, the endodermal pigment layer of the cup-ocellus forms an invaginated cup (Fig 1C and 1D), and the spot-ocelli begin to differentiate in the aboral ectoderm (Fig 1D). As the juvenile medusa grows into a sexually mature adult, the size of the rhopalium increases; thus each sensory-neuronal structure in rhopalia continues development through the juvenile stage [18].

In this work we use the emerging scyphozoan developmental model *Aurelia sp*.*1* and show that *so* and *eya* are co-expressed in developing photosensory tissues of pigment-cup ocelli, as well as in developing mechanosensory tissues and the proximal pacemaker domain of active neurogenesis in rhopalia. These observations support arguments for a shared ancestral function of these components of the RDGN, basal to the cnidarian and bilaterian clades. However, in addition to *pax6*, which is not found in Cnidaria, *dac* is not found in the *Aurelia* genome, although we found *dac* orthologs in the genomes of anthozoan cnidarians, which lack photoreceptor organs. Thus, neither *pax6* nor *dac* is required for eye development outside Bilateria. In addition, we find that neither *paxA* nor *paxB* is expressed in developing photosensory tissues of pigment-cup ocelli in *Aurelia*, suggesting that *pax* genes are evolutionarily more labile components of the RDGN controlling eye development than *so* and *eya*, at least across class-level taxa within medusozoan Cnidaria.

## Results and Discussion

### *Aurelia* genome encodes orthologs to *so*, *optix*, *eya*, *paxA* and *paxB*

**Fig 2:**
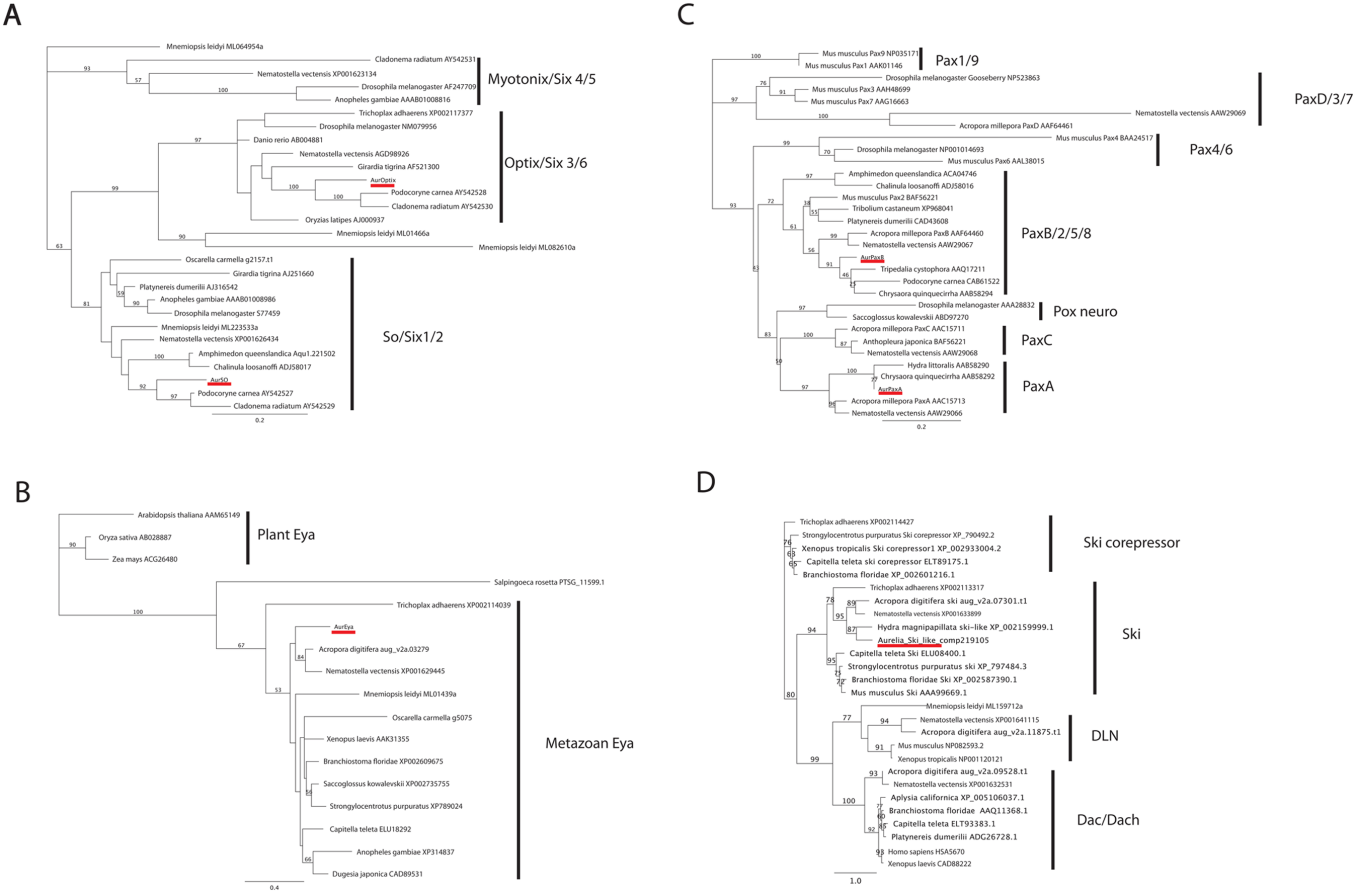
Maximum likelihood phylogenetic analyses of Six (A), Eya (B), Pax (C) and Dach (D) protein families. *Aurelia sp*.*1* sequence is highlighted in red. The metazoan Eya phylogeny (B) is rooted with plant Eya sequences. The Dach phylogeny is rooted with Ski, Ski-corepressor and DLN family sequences. Sequence IDs/accession numbers are shown with the name of each sequence. Bootstrap support values are shown at each node except when lower than 50%. The unit of the branch length is the number of substitutions per site.

We employed degenerate primers PCR and RACE to recover *Aurelia* orthologs of genes suspected to be involved in bilaterian and/or cnidarian eye development. Our initial PCR targeted the highly conserved region of each gene using degenerate primers (S1 Table) according to the previously established protocol [27]. Candidate genes were *so*, *optix*, *eya*, *dac*, *paxA* and *paxB*. Gene fragments were successfully amplified for all but *dac*. We found *dachshund*-like sequences in the genomes of anthozoan cnidarians *Nematostella vectensis* and *Acropora digitifera* via the protein BLAST search using the *Branchiostoma floridae dac* sequence (AmphiDac; NCBI accession number AAQ11368) as a query. However, we did not find evidence of *dac* in the genomes of the medusozoan cnidarian *Hydra*, the demosponge *Amphimedon queenslandica*, the ctenophore *Mnemeopsis leidyi*, or their unicellular relatives. We searched for *dac*-like sequences in *Aurelia* genomic contigs that are currently being assembled (unpublished data), as well as in developmental transcriptomes across *Aurelia* life cycle stages (planula, polyp, strobila, ephyra and medusa (Fig 1A); Gold et al., unpublished; [28, 29]). We recovered a single *dac*-like, Dach1/Ski/Sno domain-encoding gene from the *Aurelia* data, but phylogeneic analysis (Fig 2D; see below) suggests that this gene represents a *Ski* homolog, as opposed to a genuine *dac* sequence. Thus, we find no evidence that *Aurelia* genome encodes a *dac* ortholog, although it is possible that the *dac* ortholog was not recovered for technical reasons, for instance, due to the incomplete coverage of the genome. The full-length complimentary DNA sequences for *Aurelia so* (AurSO), *optix* (AurOptix), *eya* (AurEya), *paxA* (AurPaxA) and *paxB* (AurPaxB) were obtained by RACE (Rapid Amplification of cDNA ends; the SMART RACE kit, Clontech) (see S1 Table for primer sequences), and verified against our transcriptomic dataset. Their nucleotide and translated amino acid sequences are publicly available at the NCBI website under accession numbers KJ396199 (AurSO), KJ396200 (AurOptix), KJ396201 (AurEya), KJ396202 (AurPaxA), and KJ396203 (AurPaxB).

Translated amino acid sequences of the recovered genes showed residues and/or domains characteristic of proteins encoded by the target genes. AurSO and AurOptix encode the N-terminal *Six* domain and the C-terminal *Six*-type, DNA-binding homeodomain with a characteristic tetrapeptide sequence near the N-terminus of the homeodomain (ETSY for *So*, and QKTH for *Optix*; S1 Fig). AurEya encodes a C-terminal domain similar to the *Eya* domain 1 (S2 Fig), which is known to be the catalytic motif as a protein tyrosine phosphatase [30, 31], as well as the site for the protein-protein interaction with *So* in *Drosophila* [32]. AurPaxA and AurPaxB encode the N-terminal paired domain (S3 Fig). In addition, AurPaxB encodes an octapeptide characteristic of the *pax2/5/8* family, as well as the C-terminal homeodomain (S3 Fig).

We tested our sequence-similarity-based orthology hypotheses by Maximum Likelihood phylogenetic analyses, based on aligned amino acid sequences. Sequence alignment and phylogenetic analyses were performed on the Geneious (v5.1.7) platform. Alignments were largely restricted to the conserved domains—the *Six* domain and *Six*-type homeodomain for the *six* gene family, *Eya* domain 1 for the *eya* gene family, the paired domain for the *pax* gene family and the Dach1/Ski/Sno domain for the *dac* gene family and its outgroups (DLN, Ski and Ski corepressor gene families) (S1, S2, S3 and S4 Figs). The analyses strongly support the above orthology assignments (Fig 2). Thus, AurSO belongs to the metazoan *So* clade, and AurOptix belongs to the eumetazoan *Optix* clade (Fig 2A). AurEya belongs to the metazoan *Eya* clade (Fig 2B). AurPaxA belongs to the cnidarian *PaxA* clade, and AurPaxB belongs to the metazoan *PaxB* clade (Fig 2C). These gene phylogenies strongly indicate that *so*, *optix*, *eya*, *paxA* and *paxB* were present in the last common ancestor of eumetazoans. *so* and *paxB* appear metazoan specific. *eya* has a pre-metazoan, ancient eukaryotic origin [15], and *optix* appears to have evolved in Eumetazoa. The maximum likelihood tree supports the scenario where *paxA* was a product of a lineage-specific gene duplication that generated *paxA* and *paxC* early in cnidarian evolution, the ancestral form of which is orthologous to bilaterian *pox neuro*. The alternative scenario—where all three genes were present in the eumetazoan last common ancestor, followed by a loss of *paxA* and *paxC* in Bilateria, and *pox neuro* in Cnidaria—is less parsimonious than the former. In our analyses, no non-bilaterian *pax* sequences clustered with bilaterian *pax4/6*, reinforcing the notion that *pax6* is bilaterian-specific (e.g. [17]). *dac* appears to have evolved prior to the divergence of Cnidaria and Bilateria, but the lack of *dac* orthologs in *Hydra* and *Aurelia* genomes suggests that it has been lost in medusozoan cnidarian evolution (Fig 2D).

### *so* and *eya*, but not *optix*, *paxA or paxB*, are co-expressed in the ectoderm of the developing cup-ocellus in the rhopalia

**Fig 3:**
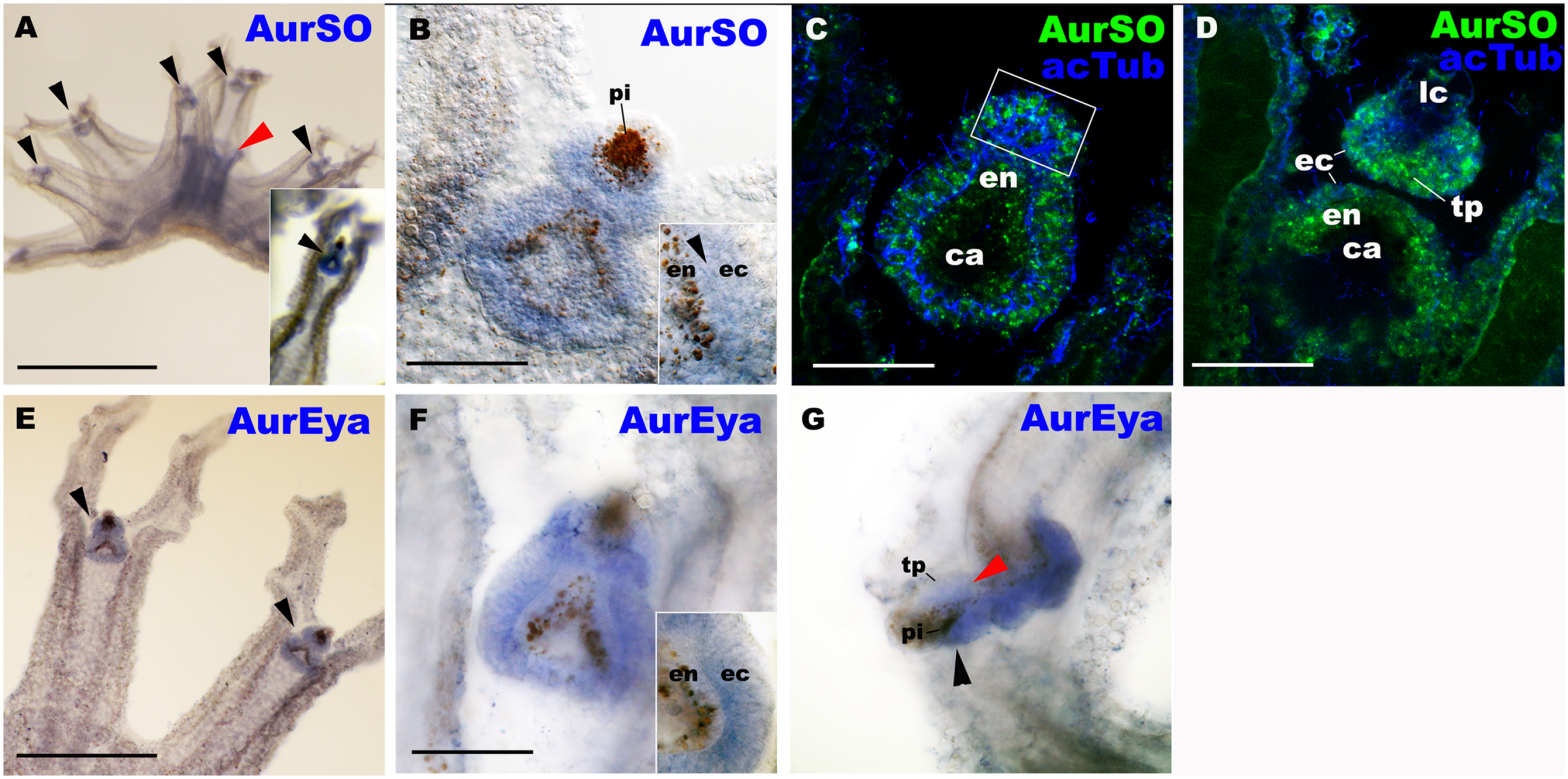
AurSO and AurEya mRNAs are co-expressed throughout the rhopalial ectoderm including the developing photosensory and mechanosensory tissues. *Aurelia sp*.*1* free-swimming ephyrae were labeled with antisense riboprobes against AurSO (A-D), and AurEya (E-G). In C and D, the rhopalia were also labeled with an antibody against acetylated ∂-tubulin (acTub). A is a lateral view of an ephyra with the mouth/manubrium facing upwards. An inset in A, and B-F are oral views with the rhopalial distal ends pointed upwards. G is a lateral view with the rhopalial distal ends pointed to the left. Note that AurSO and AurEya transcripts strongly localize to rhopalia (black arrowheads in A and E, respectively). Lower levels of manubrial expression were also detected for AurSO (red arrowhead in A), consistent with the result of the previous study [16], and for AurEya (S5 Fig). B: a medial optical section of a rhopalium. An inset shows AurSO expression in both ectoderm (ec) and endoderm (en) separated by mesoglea (arrowhead) in the proximal-lateral region of the rhopalium. C, D: confocal section of rhopalia at the plane of the developing photosensory domain (boxed) (C), and at the plane of the mechanosensory touch plate (tp; D). F: a medial optical section of a rhopalium. An inset shows AurEya expression in ectoderm (ec) but not in endoderm (en), in the proximal-lateral region of the rhopalium. G: a sagittal optical section of a rhopalium, showing AurEya expression in the developing photosensory domain (black arrowhead) and the mechanosensory touch plate (red arrowhead). Abbreviations: pi pigmented endoderm of the cup ocellus; lc lithocyst; ec ectoderm; en endoderm; ca rhopalar canal. Scale bars: 1 mm (A), 500 μm (E), 50 μm (B-D, F, G).

Next we used whole-mount *in situ* hybridization (see *Experimental procedures* for protocol) to test whether the recovered RDGN homologs were expressed in the domain of eye/cup-ocellus development at the free-swimming ephyra stage—when the cup-ocellus photoreceptor cells begin to differentiate from the ectoderm—in *Aurelia*. We found that AurSO and AurEya mRNAs were co-expressed at high levels throughout the ectoderm in rhopalia, including the ectodermal domain of cup-ocellus development (Fig 3), consistent with AurSO and AurEya functioning in development of the ectodermal photosensory tissue of the cup-ocellus in *Aurelia*. We also detected low levels of expression of both genes in the manubrium, a highly innervated feeding organ homologous to a polyp mouth (Fig 3A, S5 Fig; [16, 23]). Expression patterns of AurSO and AurEya at the late strobila stage appeared similar to those at the free-swimming ephyra stage (A-D in S6 Fig). Co-expression of AurSO and AurEya in the rhopalial ectoderm is consistent with their potential protein-protein interaction inferred from the conservation of interacting domains (see above). Co-expression of orthologs to *so* and *eya* in developing retinal tissues has been observed across Bilateria, including vertebrates [33, 34], *Drosophila* [32], and a flatworm [35], though exceptions exist (e.g. frontal eyes in amphioxus [36]). Thus there is a strong, but not perfect, correlation between those tissues expressing *so* + *eya* and retinal development in metazoans, reflecting some combination of derivation from antecedent organs [4], parallel deployment of the regulatory apparatus for eye development (e.g. [37]), and /or retention of a common prebilaterian role for these genes in photosensory system development [15].

**Fig 4:**
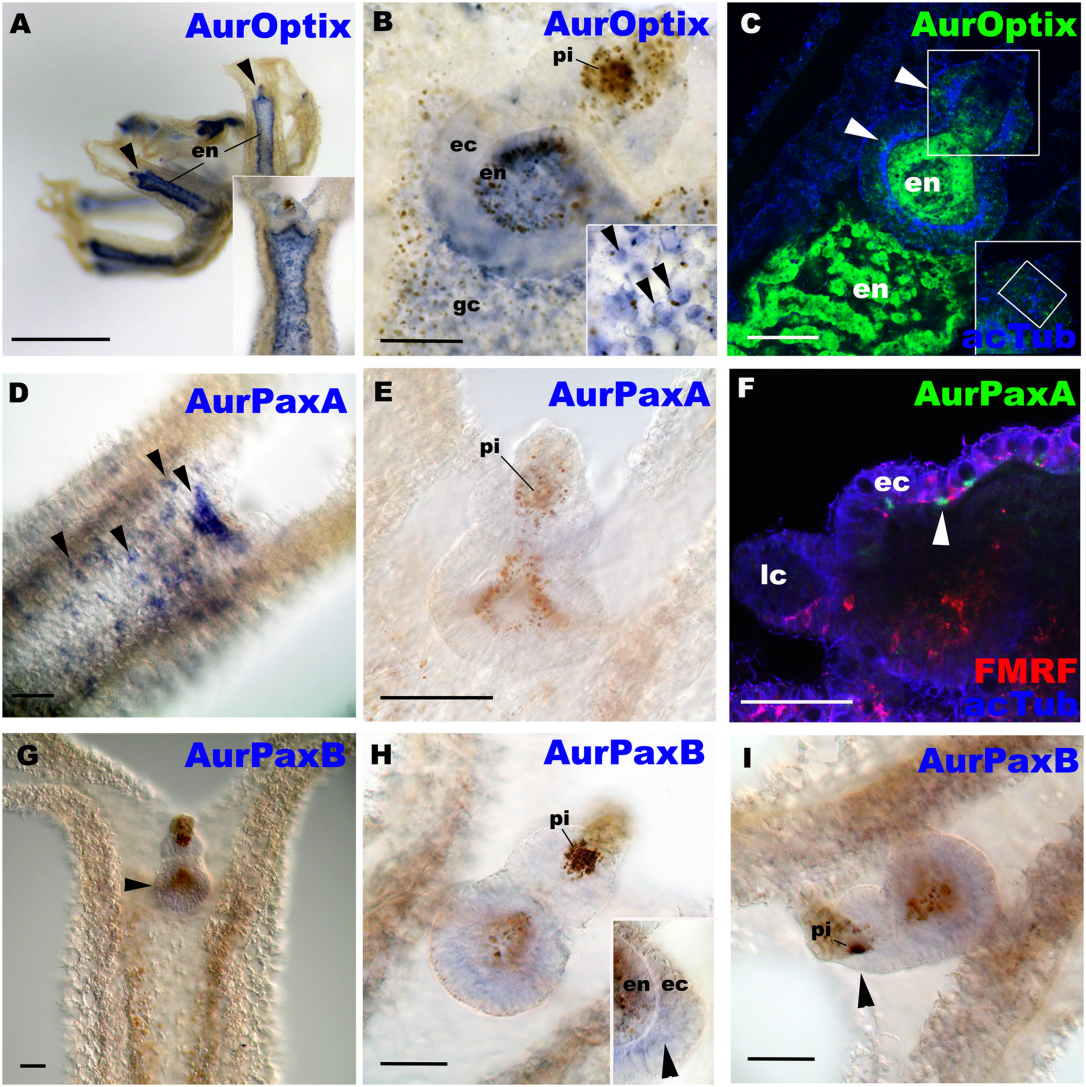
AurOptix, AurPaxA and AurPaxB mRNAs are differentially expressed outside the domain of eye development. *Aurelia sp*.*1* free-swimming ephyrae were labeled with antisense riboprobes against AurOptix (A-C), AurPaxA (D-F), and AurPaxB (G-I). A is a lateral view of an ephyra with the mouth/manubrium facing upwards. Black arrowheads show rhopalia. Note strong localization of AurOptix transcripts in the endoderm of the arms (en). In C and F, the rhopalia were also labeled with antibodies against acetylated ∂-tubulin (acTub) and FMRFamide-like neuropeptides (FMRF; in F). B, C, E, G and H, and an inset in A, are oral views with the rhopalial distal ends pointed upwards. D is an aboral view with the rhopalial distal end pointed upwards. F and I are lateral views with the rhopalial distal ends pointed to the left. B: a medial optical section of a rhopalium, showing endodermal AurOptix expression. An inset shows AurOptix-expressing endodermal pigment cells (arrowheads) in the gastrovascular canal (gc). C: a medial confocal section of a rhopalium showing AurOptix*-*expressing cells morphologically identifiable as a sensory cell (upper arrowhead) and ganglion cells (lower arrowhead) (cf. [18]). An inset is the section at the plane of the developing photosensory domain (boxed), showing little AurOptix expression. D: an optical section through the exumbrellar (aboral) ectoderm, showing strong AurPaxA expression in individual cells (arrowheads). E: a medial optical section of a rhopalium, showing no detectable levels of AurPaxA expression. F: a sagital confocal section of a rhopalium and the overlying exumbrellar ectoderm showing that *AurPaxA*-expressing cells are located at the base of the aboral ectoderm (arrowhead) in close association with the FMRFamide-immunoreactive neuronal network (FMRF). G: an oral view of a rhopalar arm. AurPaxB transcript localization in the basal region of a rhopalium is detectable at low levels (arrowhead). H: a medial optical section of a rhopalium, showing AurPaxB expression in the ectoderm (ec) of the proximal region of the rhopalium (arrowhead in an inset). I: a sagittal optical section through the pigment-cup ocellus of a rhopalium, showing little AurPaxB expression in the developing photosensory domain (arrowhead). Abbreviations: lc lithocyst. Scale bars: 1 mm (A) 50 μm (B-I).

In contrast to *so* and *eya*, *optix*, *paxA* and *paxB* mRNAs are differentially expressed outside the domain of *Aurelia* cup-ocellus development (Fig 4A–4I). Thus they do not appear to be directly involved in eye development in *Aurelia*. We found that AurOptix mRNA was expressed in a few neuronal cells in the ectoderm in rhopalia (arrowheads in Fig 4C), as well as in non-ocular pigment cells in the endoderm (Fig 4B). At the late strobila stage, AurOptix expression appeared confined to the endoderm (E and F in S6 Fig). This suggests that that AurOptix functions in the development and/or maintenance of a subset of rhopalial non-photoreceptor neurons as well as non-ocular pigment cells in the endoderm. AurPaxA-expressing cells rarely occurred in rhopalia (Fig 4D–4F). However, high levels of AurPaxA mRNA expression were detected in individual cells that were located at the base of the exumbrellar ectoderm in a close association with the FMRFamide-immunoreactive neuronal network (Fig 4F), suggesting that AurPaxA may be involved in the development of the exumbrellar nerve net. AurPaxB mRNA expression occurs in the ectoderm of the basal portion of the rhopalium (Fig 4G–4I), where a number of neurons, likely including the pacemaker neurons that receive input from photosensory cells, develop [18]. At the late strobila stage, the expression pattern of AurPaxA did not differ from that at the free-swimming ephyra stage with few cells expressing AurPaxA transcripts in developing rhopalia (G and H in S6 Fig), while we failed to detect AurPaxB transcripts at the late strobila stage (data not shown). Thus, AurPaxA and AurPaxB may function in non-ocular neural development, though AurPaxB may be important for the maintenance and/or post-strobilation development of pacemaker neurons that communicate with the photoreceptor cells in the pigment-cup eye. It is also possible that AurPaxA and B have roles in the development of non-ocular photoreceptor cells; identification of non-ocular photoreceptor cells (e.g. by localizing the expression of photosenstive molecules such as opsin in non-ocular cells) is necessary to address this possibility.

*pax6* appears to direct eye formation across bilaterian animals (except for planarians [35] and adult polychaetes [38]), and has been considered a master control gene for eye development for its ability to induce ectopic eyes in *Drosophila* (reviewed in [39]). In Cnidaria, however, orthologs to *pax6* do not exist, and cnidarian *pax* genes show little evidence for conserved roles in eye development. As mentioned, *paxA* and *paxB*, genes distantly related to *pax6* (see above), have been implicated in eye development in hydrozoan [13] and cubozoan medusae [17], respectively, while our data show no evidence that either gene is directly involved in eye development in the scyphozoan *Aurelia*. Hence, a conserved role of *pax* in eye development appears bilaterian-specific, and the function of *pax* in Cnidaria seems prone to evolutionary changes.

In addition, AurOptix does not appear to have a direct role in pigment-cup eye development in *Aurelia*, although *optix* is required for eye development in *Drosophila* [40] and vertebrates [41], and is expressed in the eye of an adult hydrozoan cnidarian [14]. Thus, in addition to differnces in pax gene orthology and function, expression domains of *optix* seem to have diverged between Cnidaria and Bilateria,. However, *optix* expression does extend into the endoderm of the rhopalia, and thus it is possible that it plays some necessary inductive-combinatorial roles in sense organ development in the overlying ectodermal sensory cell development. This could include the pigment/photosensory-cell relationship in the pigment-cup eye. We speculate that such a relationship might be comparable to the inductive interactions between germ layers in vertebrate placode development (e.g. [42, 43]).

However, co-expression of AurSO and AurEya is not restricted to the domain of eye development, but occurs throughout the rhopalial ectoderm in *Aurelia* (Fig 3), including the regions where the mechanoreceptor and the pacemaker are developing. At the strobila and the free-swimming ephyra stages, cell proliferation, sensory-neuronal differentiation, and cell-type-specific expression of neural genes *otx*, *POU-I* and *POU-IV*, occur in the rhopalial ectoderm (S7 Fig; [18, 27]). This suggests that in *Aurelia*, *so-eya* co-expression may specify local ectodermal domains of cell proliferation and sensory neurogenesis, from which sensory-neuronal structures including the eye and the mechanoreceptor develop.

### *so* and *eya*, but not *optix*, *paxA or paxB*, are upregulated during rhopalia formation

Consistent with the hypothesis that AurSO and AurEya are together involved in specifying rhopalial ectoderm, our RNA-seq-based developmental transcriptome dataset across *Aurelia* life cycle stages (Fig 1A; see Methods section for details) shows that, both AurSO and AurEya, but not AurOptix, AurPaxA or AurPaxB, are upregulated during strobilation, when rhopalial development begins (S8 Fig). In pairwise comparisons through the life cycle, all significant increases in gene expression (i.e. false discovery rate-adjusted p-values > 0.05) occurred during the early strobila to late strobila transition. Compared to the early strobila, the late strobila exhibited a 0.9 logfold increase in *AurEya*, and a 2.7 logfold increase in *AurSO*. If the polyp life stage is compared to the late strobila, these rates jump to 2.2 and 5.1 logfold increases in *AurEya* and *AurSO* respectively. In contrast, AurPaxA, AurPaxB, and AurOptix do not display a pattern of upregulation during medusa formation. However, functional analyses of AurSO and AurEya at strobilation (e.g. via RNAi; [29]) are needed to confirm and further refine the hypothesis that these genes direct rhopalial development.

### *so* and *eya* are expressed in ectodermal domains of active cell proliferation and sensory neurogenesis across Eumetazoa

In hydrozoan cnidarians, *so* and *eya* are also expressed in local sensory-neuronal structures of medusae—regardless of the specific sensory function—where cell proliferation and differentiation are active. In the medusae of *Cladonema radiatum*, a hydrozoan cnidarian, *so* and *eya* are expressed in the retinal tissues located in the tentacle bulb, a local bulge of tissues at the base of the tentacle, as well as the surrounding ectoderm [14, 15]). Hydrozoan tentacle bulbs are highly innervated, as they contain “tentacular ganglia” [44], and can develop eyes—as in the case of *C*. *radiatum*—or mechanosensory statocysts. *so* expression has been reported in tentacle bulbs with statocysts (*Craspedacusta sowerbyi* [45]), as well as in tentacle bulbs of taxa that lack eyes or statocysts (*Podocoryne carnea* [14]), indicating that *so* expression is conserved in tentacle bulbs and is not specific to eyes. In addition, the ectoderm in eye- and statocyst-less tentacle bulbs is highly active in cell proliferation and differentiation, particularly of stinging cells, the cnidocytes (as reported in *Clytia hemisphaerica* [46]). Cnidocytes may have some evolutionary relationship to sensory neurons (see [46] for summary of current evidence).

Active cell proliferation and sensory-neuronal differentiation typify ectodermal *so-eya* co-expression domains in Bilateria as well. Examples include vertebrate cranial placodes that give rise to cephalic sensory organs and ganglia (reviewed in [43]), the larval chemosensory and neurosecretory structure known as the preoral organ (a putative adenohypophysis homologue) in the cephalochordate *Branchiostoma floridae* [36], and the eye-imaginal disc that generates compound eyes in *Drosophila* (reviewed in [47]). Taken together, sensory structures with different sensory modalities appear to develop, and have evolved, within the *so* and *eya* co-expression domains characterized by local cell proliferation and sensory neurogenesis across Cnidaria and Bilateria. This suggests that in the last common ancestor to Cnidaria and Bilateria, *so-eya* co-expression within local ectodermal domains might have regulated cell proliferation and sensory-neuronal differentiation, potentially in a dose-dependent manner (e.g. *Xenopus* cranial placodes; [48]), to generate sensory structures within which different sensory modalities evolved in different lineages.

In addition, the *so-eya* ectodermal co-expression domains often occur within broad *otx* expression domains, as is the case in *Aurelia* rhopalia [27] and in the cephalar sensory organization in Bilateria [49]. These observations taken together are consistent with the hypothesis that an ancestral regulatory nexus consisting of *otx-so-eya* controlled neurosensory localization prior to the cnidarian-bilaterian split. This nexus would then presumably have been subject to considerable evolutionary modifications of sense organ specification in both cnidarian and bilaterian lineages.

It is unclear whether our model of sensory organ evolution can be generalized across all animals. Discrete sensory structures are found outside the Cnidaria and Bilateria. For instance, a gravity-sensitive statocyst, consisting of statolith-containing lithocytes supported by four groups of ciliated cells (balancers), occurs in the sensory structure complex (“apical organ”) at the aboral pole of ctenophores [50]. Some sponge larvae develop a ring of ciliated cells around the posterior pole (in terms of swimming direction) that are responsive to blue light and are used for steering the animal [51]. The genomes of the ctenophore *Mnemiopsis leidyi* and sponges (e.g. *Amphimedon queenslandica*, *Sycon ciliatum*, and *Oscarella sp*.) encode *so*, *eya* and *pax* (A and B in *M*. *leidyi*; B in sponges) (this study, [16, 52–55]). In these non-eumetazoan animal taxa, however, detailed analyses of developmental expression and functions of these genes in relation to sensory structure development, or the functional characterization of cells or structures that express these genes (e.g. larval “sensory” cells in the calcareous sponge *S*. *ciliatum* [55]), are lacking. These data will be necessary to elucidate whether the function of *so-eya* in specifying neurosensory domains has an ancient evolutionary origin predating the divergence of Eumetazoa.

## Conclusions

Here we reported that in the scyphozoan *Aurelia*, developing ectodermal retinal tissue co-expressed *so* and *eya*, but not *optix*, *paxA* or *paxB*, suggesting that *pax* and *optix* genes have not imposed strong constraints in eye evolution beyond Bilateria. In addition, the complex distribution of sensory structures in the *so-eya* expressing field (discussed above) combined with our new observations in *Aurelia* suggest repeated evolutionary gain, or loss of eyes, or possibly sense organ type conversions within the *so* and *eya* expression domains in Cnidaria.

## Materials and Methods

### Animals and fixation

Strobilae, ephyrae and metephyrae of *Aurelia sp*.*1* (*sensu* [27]) were obtained from the Cabrillo aquarium (San Pedro, CA). Animals were fixed as previously described [27].

### Nucleic acid extraction and cDNA synthesis

Genomic DNA and total RNA were simultaneously extracted according to the published protocol [56]. First-strand cDNAs were synthesized by using SuperScript III First-Strand Synthesis System for RT-PCR (Invitrogen) or BD SMART RACE cDNA Amplification Kit (BD Biosciences).

### Degenerate PCR, RACE, cloning and sequencing

Homologous sequences to *so*, *eya*, *optix*, *paxA* and *paxB* genes were recovered from the *A*. *sp*.*1* genome via PCR with degenerate primers, using polyp and ephyra cDNAs as the PCR templates. Sequences of the 5’ and 3’ regions of genes of interest were obtained via RACE using ephyra cDNAs as templates. PCR products were cloned into the pCRII-TOPO vector using the TOPO TA cloning Dual Promotor kit (Invitrogen) and sequenced at the UCLA Genotyping and Sequencing Core facility. The alignment of the sequences and assembly of contigs were performed using the CodonCode Aligner (v.1.5.2, CodonCode Corporation).

### Phylogenetic analyses

Sequence alignment and phylogenetic analyses were performed on the Geneious platform (v.5.1.7). Related sequences were retrieved via the protein BLAST search using the *Aurelia* sequence as queries, from GenBank at the NCBI website (http://blast.ncbi.nlm.nih.gov/Blast.cgi), *Acropora digitifera* genome (Version 1.1) portal at the OIST Marine Genomics unit (*Acropora digitifera* sequence; http://marinegenomics.oist.jp/genomes/viewer?project_id=3&current_assembly_version=oist_v1.1), *Mnemiopsis* genome project portal at National Human Genome Research Institute (*Mnemiopsis leidyi* sequences; http://research.nhgri.nih.gov/mnemiopsis/), Compagen at the Bosch Laboratory at the University of Kiel (*Oscarella* sequences; http://compagen.zoologie.uni-kiel.de/), and Origins of Multicellularity Database at the Broad Institute (*Salpingoeca rosetta* sequnce; http://www.broadinstitute.org/annotation/genome/multicellularity_project/MultiHome.html). Peptide sequences were aligned with MUSCLE (v3.7) [57] configured for highest accuracy (MUSCLE with default settings). After alignment, ambiguous regions (i.e. containing gaps and/or poorly aligned) were manually removed. Phylogenetic trees were reconstructed using the maximum likelihood method implemented in the PhyML program [58]. The WAG substitution model [59] was selected assuming an estimated proportion of invariant sites and 4 gamma-distributed rate categories to account for rate heterogeneity across sites. The gamma shape parameter was estimated directly from the data. Reliability for internal branches of maximum likelihood trees was assessed using the bootstrapping method (100 bootstrap replicates).

### Immunohistochemistry and confocal microscopy

Immunohistochemistry was performed as previously described [60]. Primary antibodies that were used for this study were reactive against FMRFamide (“FMRF”; rabbit, 1:500 dilution; US Biological), GLWamide (“GLW”; rabbit 1676 IIIp, 1:300, provided by Dr. T. Leitz; for detailed information for this antibody, see [61]), Acetylated ∂-Tubulin (“acTub”; mouse, 1:1000; Sigma), Tyrosinated ∂-Tubulin (“tyrTub”; mouse, 1:1000; Sigma) and Phosphorylated Histone H3 (“H3”; rabbit, 1:1000; Abcam). The anti-FMRFamide antibody is assumed to react with endogenous neuropeptides with the C-terminal sequence Gly-Arg-Phe-NH_2_ in cnidarians, and the anti-GLWamide antibody reacts with neuropeptides with the C-terminal sequence Gly-Leu-Trp-NH_2_ [61, 62]. In *Aurelia*, the anti-FMRFamide labels the ectodermal sensory nerve net (known as the “diffuse nerve net”, or DNN) and bilaterally arranged groups of ectodermal sensory neurons and photosensory cells in rhopalia, while the anti-GLWamide labels a subset of ectodermal sensory neurons in the intermediate-proximal region of the rhopalia [18]; differences in immunoreactivity patterns in these antibodies presumably reflect differences in the spatial distribution of cells expressing antigens to respective antibodies. Secondary antibodies that were used for this study were AlexaFluor 488 (mouse, 1:200; Molecular Probes), AlexaFluor 568 (rabbit, 1:200; Molecular Probes), AlexaFluor 633 (mouse, 1:200; Molecular probe) and Cy5 (rabbit, 1:200; Jackson Laboratory).

### *in situ* hybridization

Probe template preparation and fluorescent *in situ* hybridization were conducted as described previously [27]. Primers used are listed in S1 Table. The DNA templates were the 819 bp fragment encompassing the Six domain and a part of 3’UTR for AurSO, the 281 bp fragment at the 3’ UTR region for AurEya, and 3’RACE products AurOptix (≈ 480 bp), AurPaxA (≈ 913 bp) and AurPaxB (≈ 1085 bp). For detection of RNA probes by alkaline phosphatase-NBT/BCIP colorimetric reaction, the following modifications were made after probe hybridization and the subsequent washes with decreasing concentration of hybridization buffer. Specimens underwent a series of washes in 0.05x SSC/PBSTr solutions (PBSTr; 25%, 50%, 75% and 100%) each for 10 minutes at RT, followed by a wash in PBSTr for one hour at RT. The samples were blocked in 10% normal goat serum and 1% bovine serum albumin in PBSTr for one hour, and were then incubated with anti-digoxigenin-alkaline-phosphatase-conjugated antibody (Roche; 1:2000) for four hours at RT. The antibody was washed in PBSTr overnight at RT. The specimens were transferred to the color reaction buffer (100 mM Tris pH9.5, 100 mM NaCl, 50 mM MgCl_2_, 1 mM lavamisol, 0.1% Tween 20), and NBT/BCIP (4.5/3.5 μl/ml; Boehringer Mannheim) was added as the enzyme substrate. The color reaction was stopped by rinsing in PBSTr, followed by the ethanol series, incubation in methanol and rehydration in water. The specimens were mounted in 70% glycerol in water.

### RNA-Seq and Gene Expression Analyses

To generate the transcriptome paired-end cDNA libraries for seven life stages (early planula larva, late planula larva, polyp, early strobila, late strobila, ephyra, and juvenile medusa) were generated using the TruSeq RNA Sample Prep Kit (v2 Illumina). The cDNA libraries were run on three lanes of an Illumina Hi-Seq, generating a100 base pair paired-end dataset. Reads with a FastQ quality score less than 20 were removed using the Filter FastQ tool in Galaxy. ∼320,000,000 100 base pair paired-end reads were assembled into predicted transcripts *de novo* using the Trinity software package [63].

Biological replicates were generated for the life stages, resulting in three biological replicates per stage, except for the “juvenile” and “early planula” stages, which had two replicates each. Biological replicates were sequenced using 50 base pair single end reads. The forward reads from the original 100 paired end data and the biological replicates were mapped back to the transcriptome, and abundance estimates for raw counts were calculated using the RSEM package [64]. Pairwise comparisons between different life stages were performed using the EdgeR package [65] included in Trinity. Significant changes in gene expression were identified using a false discovery rate (FDR) adjusted p-value cutoff of 0.05 for each pairwise comparison performed in EdgeR. Detailed methods regarding library preparation and downstream analyses will be included in an upcoming publication (Gold et al. in prep).

## Supporting Information

S1 Fig Six protein sequence alignment. A protein sequence alignment with selected taxa. The black line indicates the sites that correspond to the Six domain (SD), and the blue line indicates the sites that correspond to the homeodomain (HD). The green line indicates the sites that were used for phylogenetic analyses. Tetrapeptide sequences diagnostic of each subfamily are boxed in red.
(TIF)

S2 Fig Eya protein sequence alignment. A protein sequence alignment with selected taxa. The black line indicates the sites that correspond to the Eya domain 1. The green line indicates the sites that were used for phylogenetic analyses.
(TIF)

S3 Fig Pax protein sequence alignment. A protein sequence alignment with selected taxa. The black line indicates the sites that correspond to the paired domain (PD), and the blue line indicates the sites that correspond to the homeodomain (HD) for boxed sequences. The green line indicates the sites that were used for phylogenetic analyses. Octapeptide sequences diagnostic of Pax2/5/8/B are boxed in red.
(TIF)

S4 Fig Dachshund/Dach protein sequence alignment. A protein sequence alignment with selected taxa. The black line indicates the sites that correspond to the Dachbox-N domain, and the Dachbox-C domain is boxed in blue; *Acropora* Dachbox-C domain could not be unambiguously aligned. The green line indicates the sites that were used for phylogenetic analyses.
(TIF)

S5 Fig AurEya transcripts are detectable at low levels in the manubrium at the ephyra stage in *Aurelia sp*.*1*. *Aurelia sp*.*1* free-swimming ephyrae were labeled with an antisense riboprobe against AurEya. A lateral view of the manubrium. The tip of the manubrium is pointed to the left. Arrowhead in an inset shows endodermal expression of AurEya in the boxed region. Scale bar: 100 μm.
(TIF)

S6 Fig mRNA expression patterns of AurSO, AurEya, AurOptix and AurPaxA at the late strobila stage in *Aurelia sp*.*1*. *Aurelia sp*.*1* late strobilae were labeled with antisense riboprobes against AurSO (A, B), AurEya (C, D), AurOptix (E, F) and AurPaxA (G, H). In G and H, the strobila was also labeled with an antibody against acetylated ∂-tubulin (acTub). Following staining, interconnected segments of developing ephyrae in the strobila (a “prephyra”) were separated by severing the longitudinal muscle fibers linking them, in order to facilitate imaging. A, C and E show oral views of prephyrae, and B, D and F show close-up images of rhopalia viewed from the oral side. Arrowheads in A and C show rhopalia. Note strong transcript localization of AurSO and AurEya in the rhopalial ectoderm including the region that develops photoreceptors (arrowheads in B and D). An arrowhead in F shows endodermal expression of AurOptix in a rhopalium. G shows confocal sections through the rhopalium showing the lack of AurPaxA-expressing cells in the region that develops a pigment-cup ocellus (arrowhead). H shows a rare AurPaxA-expressing cell in the endoderm (en) of a rhopalium (arrowhead). Scale bar: 500 μm (A, C, E), 50 μm (B, D, F-H).
(TIF)

S7 Fig Active cell proliferation occurs in the pseudostratified ectoderm of developing rhopalia at the late strobila and free-swimming ephyra stages in *Aurelia sp*.*1*. *Aurelia sp*.*1* late strobilae (A) and free-swimming ephyrae (B, C) were labeled with antibodies against Tyrosinated ∂-Tubulin (tyrTub) and Phosphorylated Histone H3 (H3), a mitotic marker. A: confocal sections through a rhopalium at the late strobila stage showing numerous mitotic figures in the rhopalial ectoderm (arrowheads). B: medial-to-superficial confocal sections through the oral region of the rhopalium in a free-swimming ephyra, partially exposing the endoderm (en). Distal side is up, viewed orally. White arrowheads show apically localized mitotic figures in the ectoderm in intermediate (in) and basal (ba) segments, while a blue arrowhead indicates a mitotic figure in the endoderm. C: confocal sections through the ectodermal epithelium of the proximal region of the rhopalium in a free-swimming ephyra. Apical side is up, basal side down. Note that the mitotic cell is positioned apically and the plane of cell division is perpendicular to the epithelial surface as indicated by the orientation of mitotic spindles (ms), a pattern typical of mitosis in pseudostratified epithelia (e.g. the vertebrate neural tube; reviewed in [66]). Nuclei are labeled with the fluorescent dye TOTO. Abbreviations: lc lithocyst; te terminal segment. Scale bars: 50 μm (A, B), 10 μm (C).
(TIF)

S8 Fig Differential expression of RDGN genes across *Aurelia* life cycle stages. Transcript levels are normalized by Transcripts per Million (TPM). Significant changes in gene expression (defined as false discovery rate (FDR) adjusted p-values < 0.05 in EdgeR pairwise comparisons) are noted with an asterisk. Note that AurSO and AurEya are upregulated at the strobila life stage, and that this expression level is maintained or increased through the development of the medusa. This is consistent with the hypothesis that these genes play a role in rhopalium development. Conversely, AurOptix, AurPaxA, and AurPaxB fail to exhibit any sustained pattern of differential expression through the life cycle, suggesting they play more general roles in *Aurelia’s* development.
(TIF)

S9 Fig Edge R output showing the results of differential gene expression analysis for *Aurelia* RDGN genes. (XLSX)

S10 Fig Whole-mount fluorescent *in situ* hybridization with an AurSO antisense probe specifically labels rhopalia. Confocal sections through rhopalia (rh) in *Aurelia sp*.*1* ephyrae fluorescently labeled with an antisense riboprobe against AurSO (A), and with an AurSO sense riboprobe (B) as a control. The specimens are viewed orally, and the distal side is up. The rhopalia are outlined in white. Strong labeling occurs in rhopalia (rh) when an antisense probe is used (A), but not when a sense probe is used (B). Scale bars: 100 μm.
(TIF)

S1 Table List of primers used in this study. (XLS)
