# Supplementary figures and images for: Gene Expression Data from the Moon Jelly, Aurelia, Provide Insights into the Evolution of the Combinatorial Code Controlling Animal Sense Organ Development
Source: PLoS One. 2015 Jul 30;10(7):e0132544. doi: 10.1371/journal.pone.0132544 (PMC4520661; doi:10.1371/journal.pone.0132544)

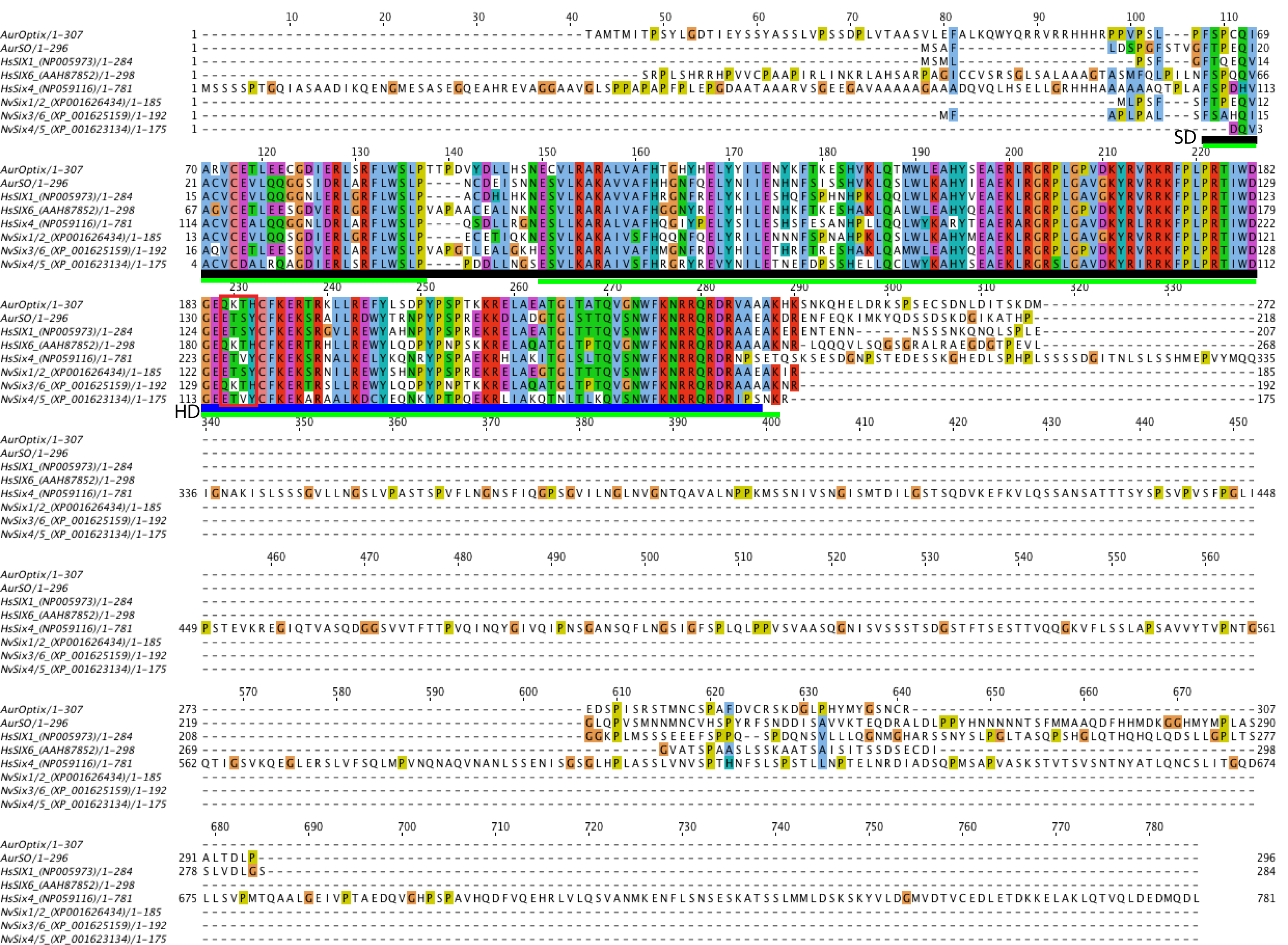

Supplement: S1 Fig — A protein sequence alignment with selected taxa. The black line indicates the sites that correspond to the Six domain (SD), and the blue line indicates the sites that correspond to the homeodomain (HD). The green line indicates the sites that were used for phylogenetic analyses. Tetrapeptide sequences diagnostic of each subfamily are boxed in red. (TIF) [file pone.0132544.s001.tif]

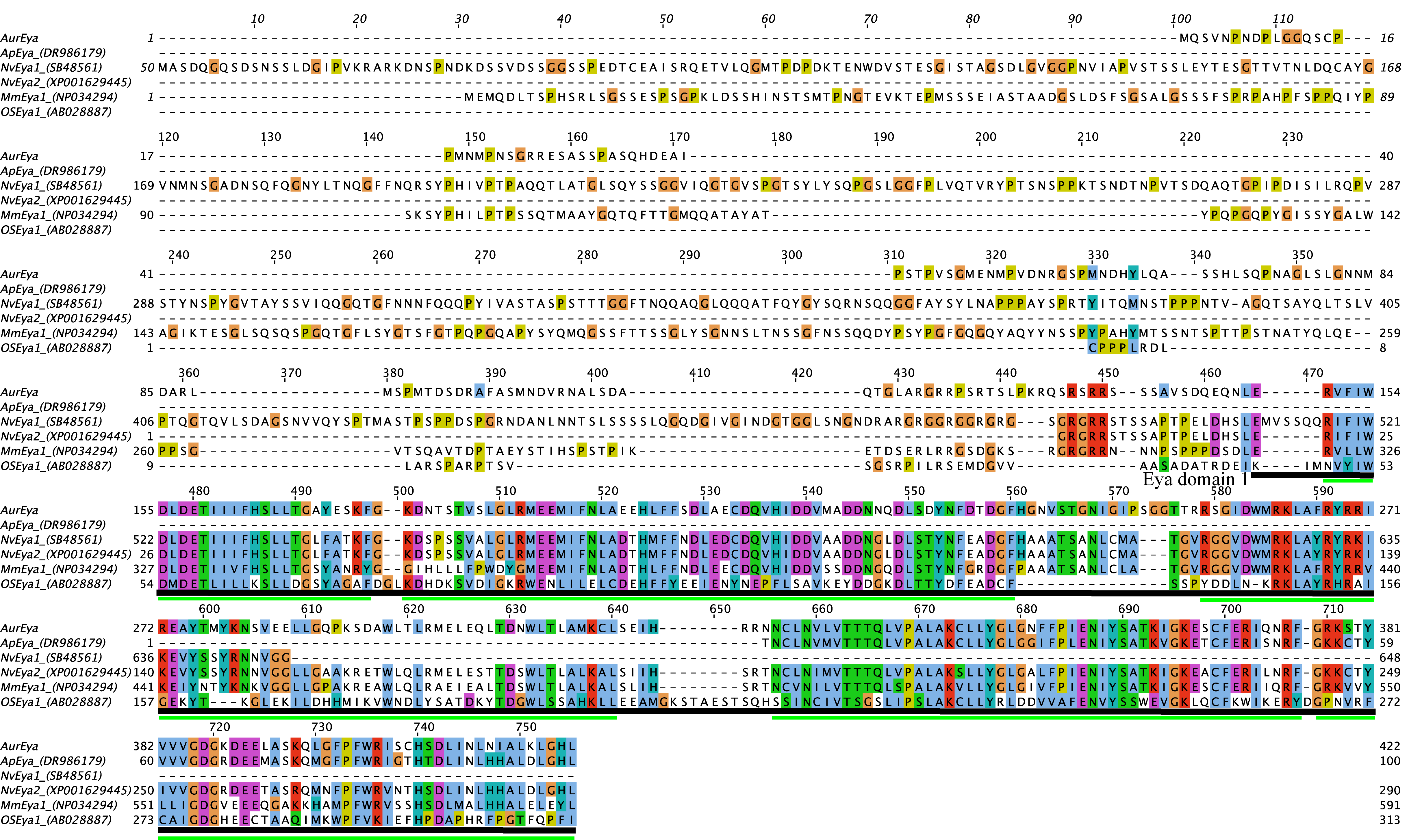

Supplement: S2 Fig — A protein sequence alignment with selected taxa. The black line indicates the sites that correspond to the Eya domain 1. The green line indicates the sites that were used for phylogenetic analyses. (TIF) [file pone.0132544.s002.tif]

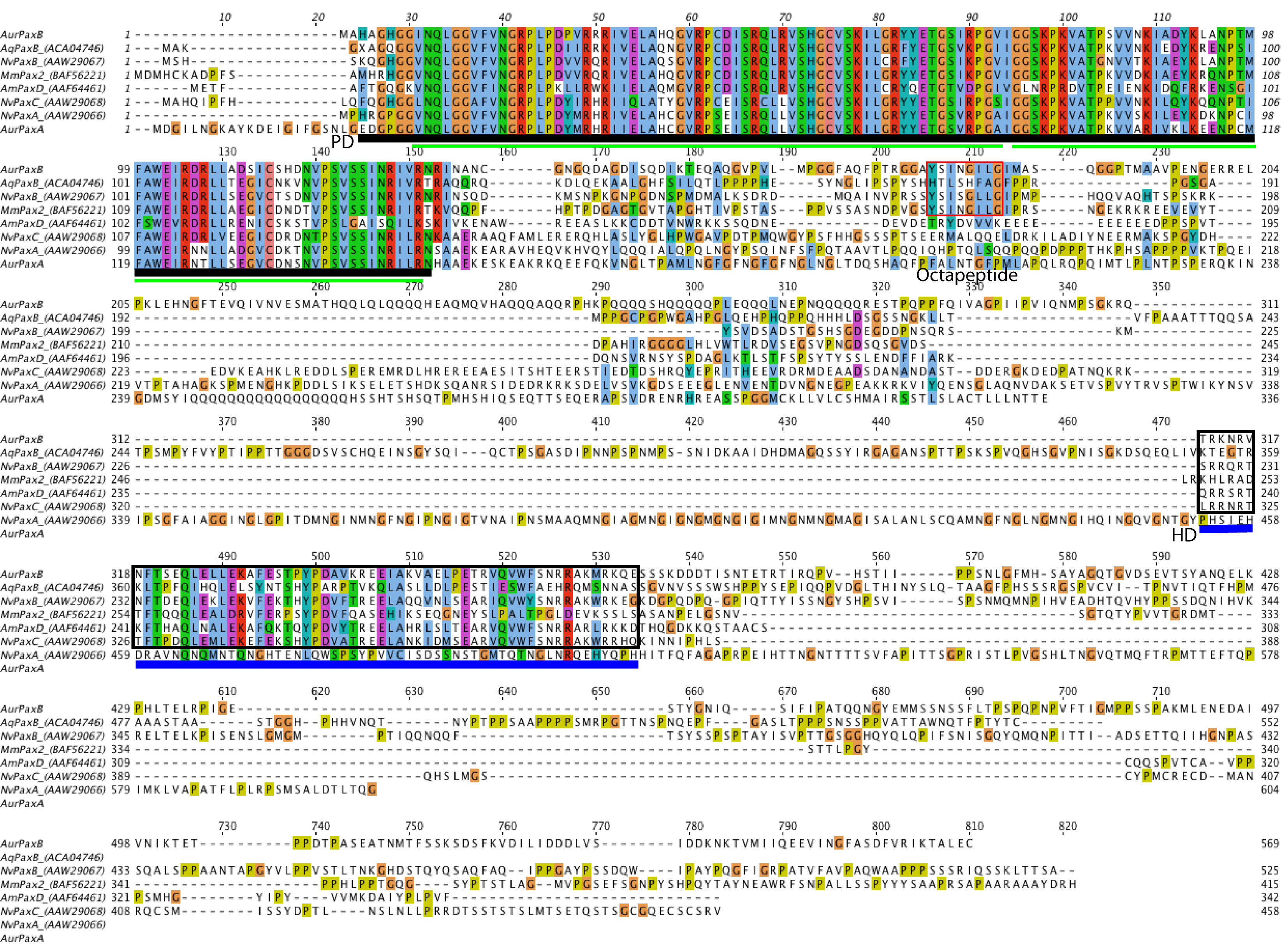

Supplement: S3 Fig — A protein sequence alignment with selected taxa. The black line indicates the sites that correspond to the paired domain (PD), and the blue line indicates the sites that correspond to the homeodomain (HD) for boxed sequences. The green line indicates the sites that were used for phylogenetic analyses. Octapeptide sequences diagnostic of Pax2/5/8/B are boxed in red. (TIF) [file pone.0132544.s003.tif]

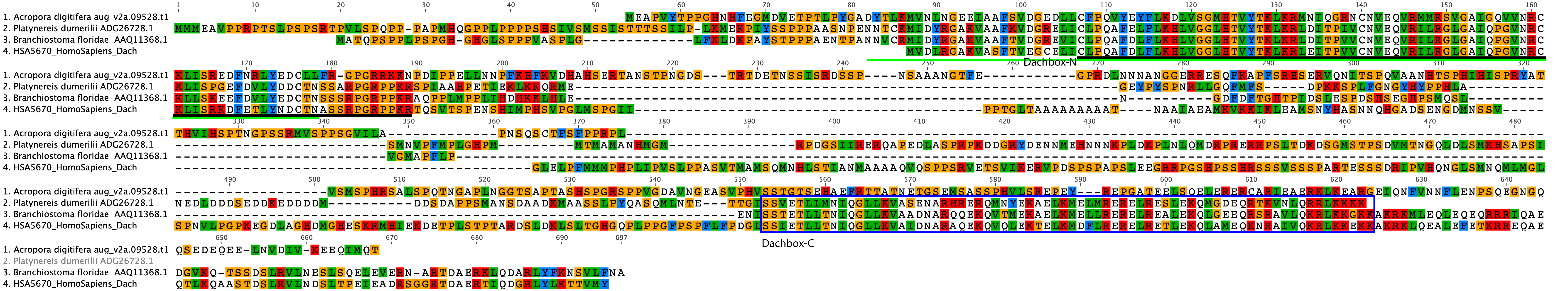

Supplement: S4 Fig — A protein sequence alignment with selected taxa. The black line indicates the sites that correspond to the Dachbox-N domain, and the Dachbox-C domain is boxed in blue; Acropora Dachbox-C domain could not be unambiguously aligned. The green line indicates the sites that were used for phylogenetic analyses. (TIF) [file pone.0132544.s004.tif]

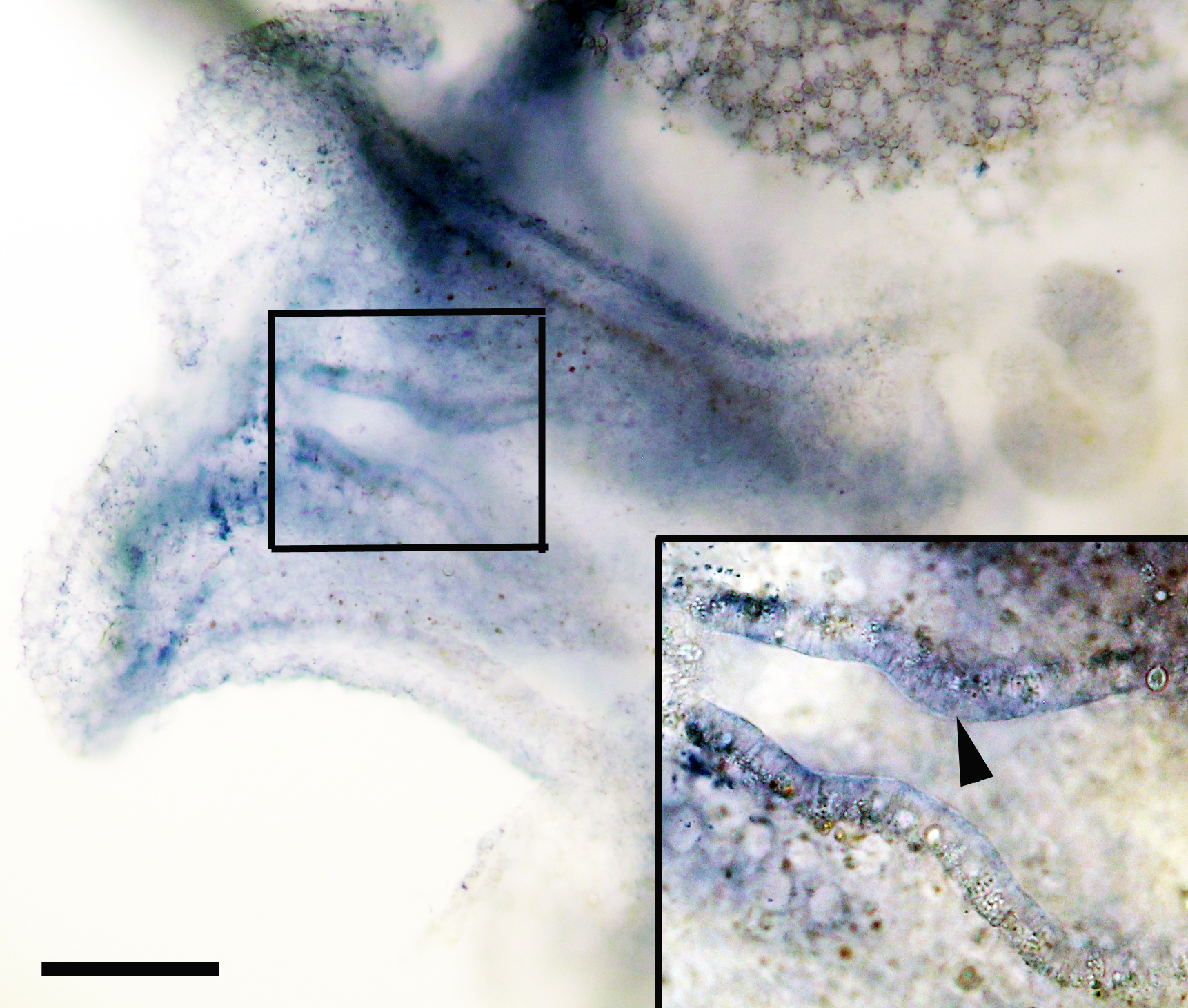

Supplement: S5 Fig — Aurelia sp.1 free-swimming ephyrae were labeled with an antisense riboprobe against AurEya. A lateral view of the manubrium. The tip of the manubrium is pointed to the left. Arrowhead in an inset shows endodermal expression of AurEya in the boxed region. Scale bar: 100 μm. (TIF) [file pone.0132544.s005.tif]

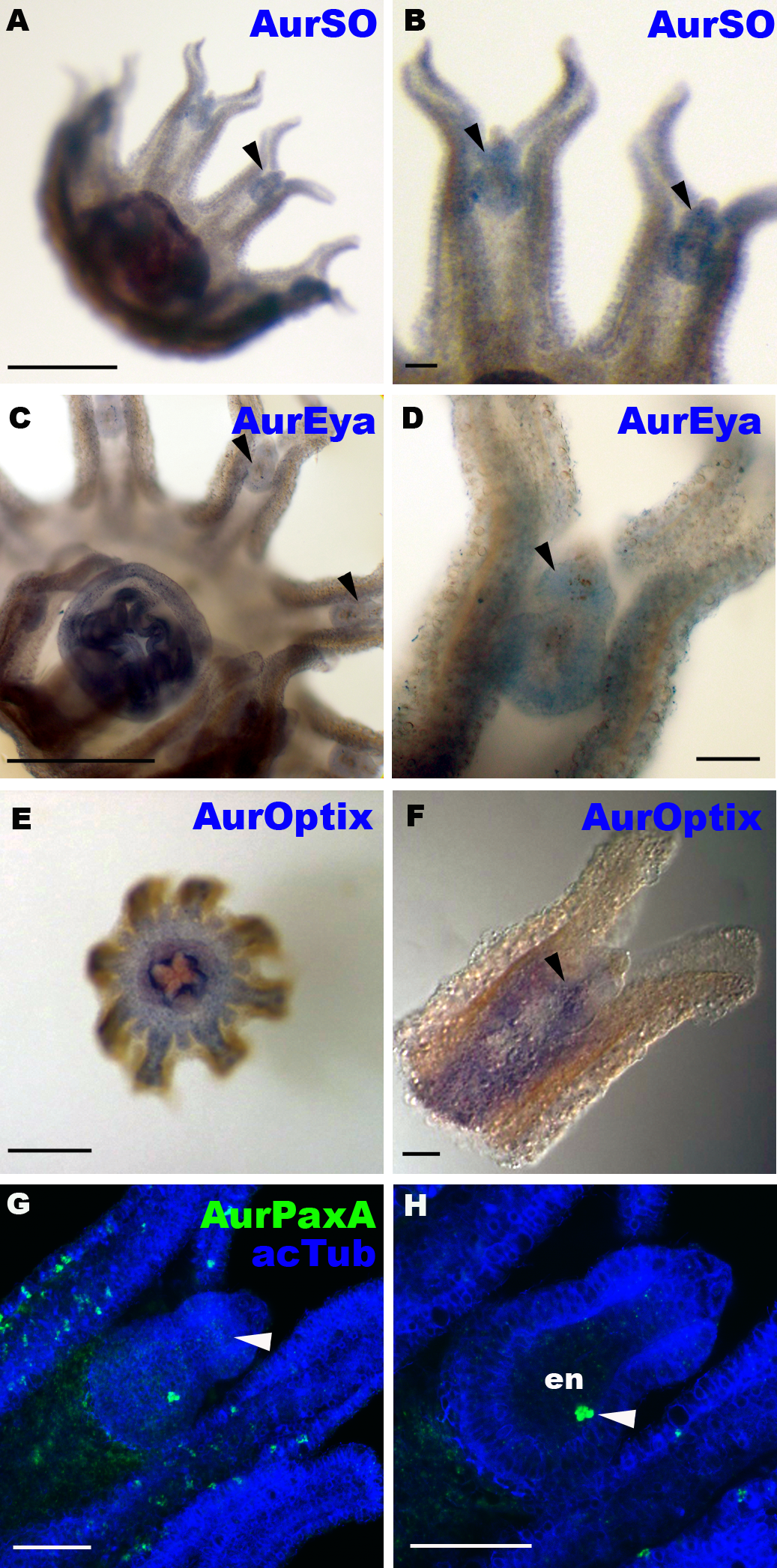

Supplement: S6 Fig — Aurelia sp.1 late strobilae were labeled with antisense riboprobes against AurSO (A, B), AurEya (C, D), AurOptix (E, F) and AurPaxA (G, H). In G and H, the strobila was also labeled with an antibody against acetylated ∂-tubulin (acTub). Following staining, interconnected segments of developing ephyrae in the strobila (a “prephyra”) were separated by severing the longitudinal muscle fibers linking them, in order to facilitate imaging. A, C and E show oral views of prephyrae, and B, D and F show close-up images of rhopalia viewed from the oral side. Arrowheads in A and C show rhopalia. Note strong transcript localization of AurSO and AurEya in the rhopalial ectoderm including the region that develops photoreceptors (arrowheads in B and D). An arrowhead in F shows endodermal expression of AurOptix in a rhopalium. G shows confocal sections through the rhopalium showing the lack of AurPaxA-expressing cells in the region that develops a pigment-cup ocellus (arrowhead). H shows a rare AurPaxA-expressing cell in the endoderm (en) of a rhopalium (arrowhead). Scale bar: 500 μm (A, C, E), 50 μm (B, D, F-H). (TIF) [file pone.0132544.s006.tif]

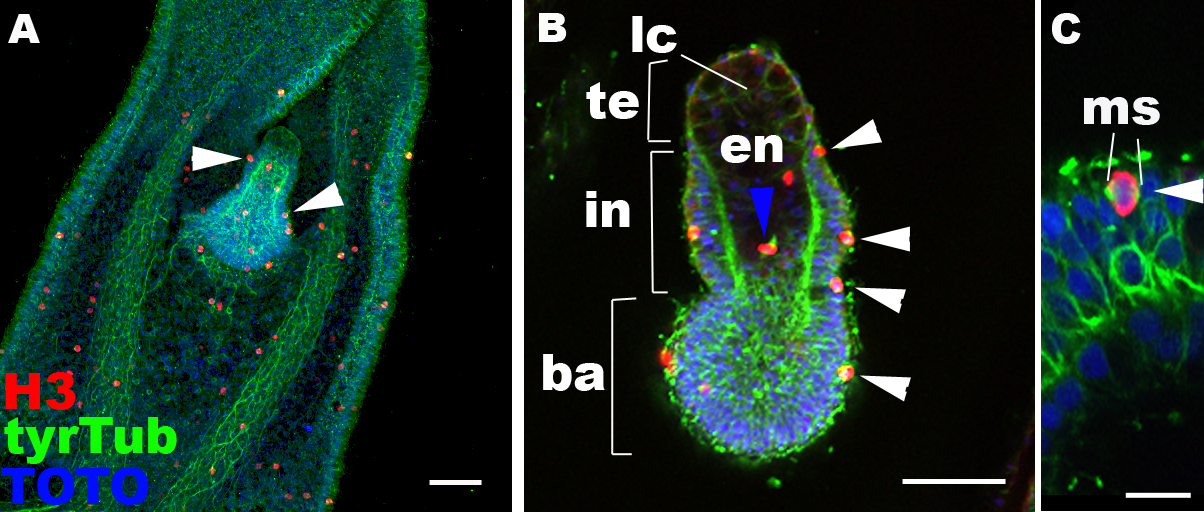

Supplement: S7 Fig — Aurelia sp.1 late strobilae (A) and free-swimming ephyrae (B, C) were labeled with antibodies against Tyrosinated ∂-Tubulin (tyrTub) and Phosphorylated Histone H3 (H3), a mitotic marker. A: confocal sections through a rhopalium at the late strobila stage showing numerous mitotic figures in the rhopalial ectoderm (arrowheads). B: medial-to-superficial confocal sections through the oral region of the rhopalium in a free-swimming ephyra, partially exposing the endoderm (en). Distal side is up, viewed orally. White arrowheads show apically localized mitotic figures in the ectoderm in intermediate (in) and basal (ba) segments, while a blue arrowhead indicates a mitotic figure in the endoderm. C: confocal sections through the ectodermal epithelium of the proximal region of the rhopalium in a free-swimming ephyra. Apical side is up, basal side down. Note that the mitotic cell is positioned apically and the plane of cell division is perpendicular to the epithelial surface as indicated by the orientation of mitotic spindles (ms), a pattern typical of mitosis in pseudostratified epithelia (e.g. the vertebrate neural tube; reviewed in [66]). Nuclei are labeled with the fluorescent dye TOTO. Abbreviations: lc lithocyst; te terminal segment. Scale bars: 50 μm (A, B), 10 μm (C). (TIF) [file pone.0132544.s007.tif]

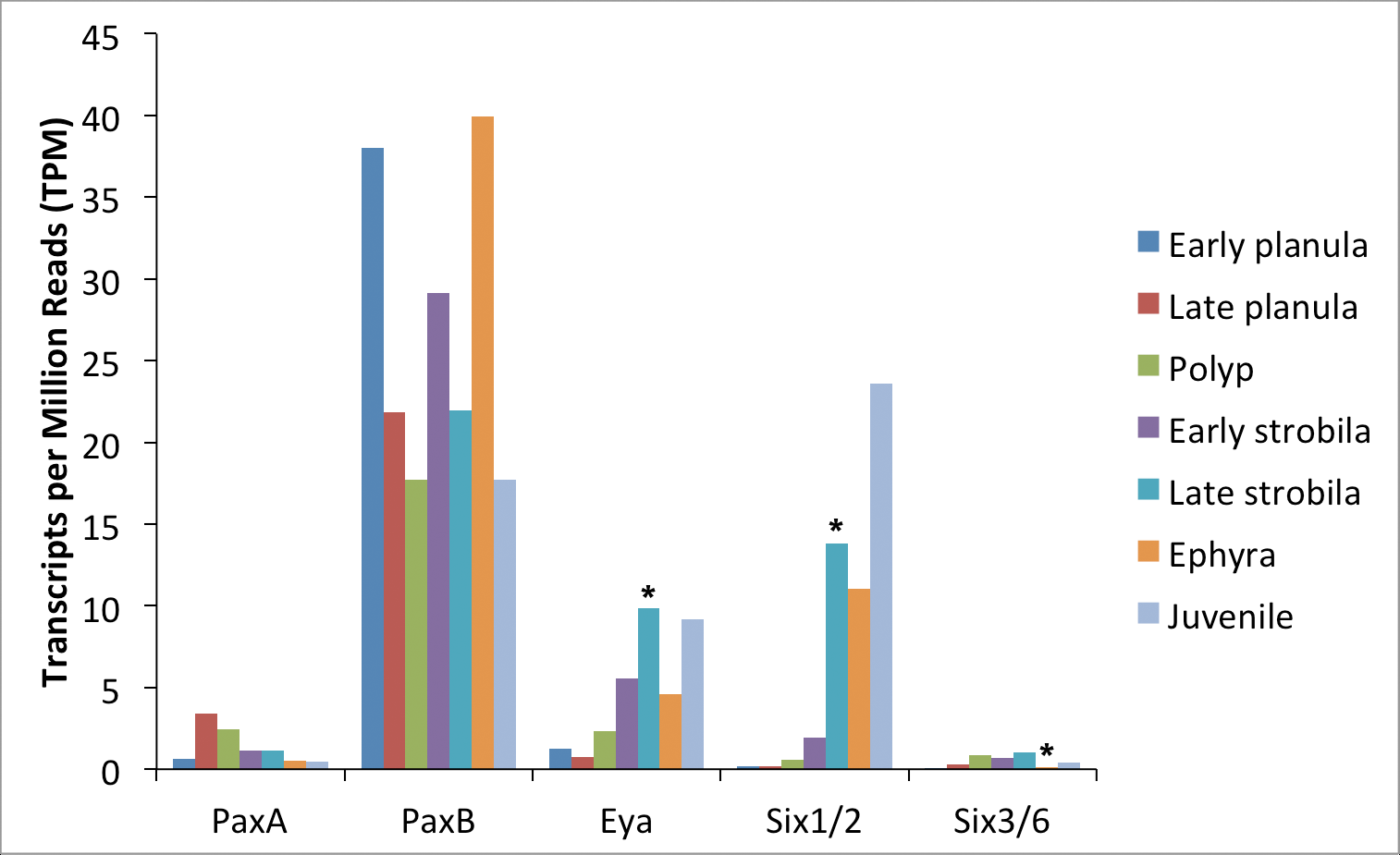

Supplement: S8 Fig — Transcript levels are normalized by Transcripts per Million (TPM). Significant changes in gene expression (defined as false discovery rate (FDR) adjusted p-values < 0.05 in EdgeR pairwise comparisons) are noted with an asterisk. Note that AurSO and AurEya are upregulated at the strobila life stage, and that this expression level is maintained or increased through the development of the medusa. This is consistent with the hypothesis that these genes play a role in rhopalium development. Conversely, AurOptix, AurPaxA, and AurPaxB fail to exhibit any sustained pattern of differential expression through the life cycle, suggesting they play more general roles in Aurelia’s development. (TIF) [file pone.0132544.s008.tif]

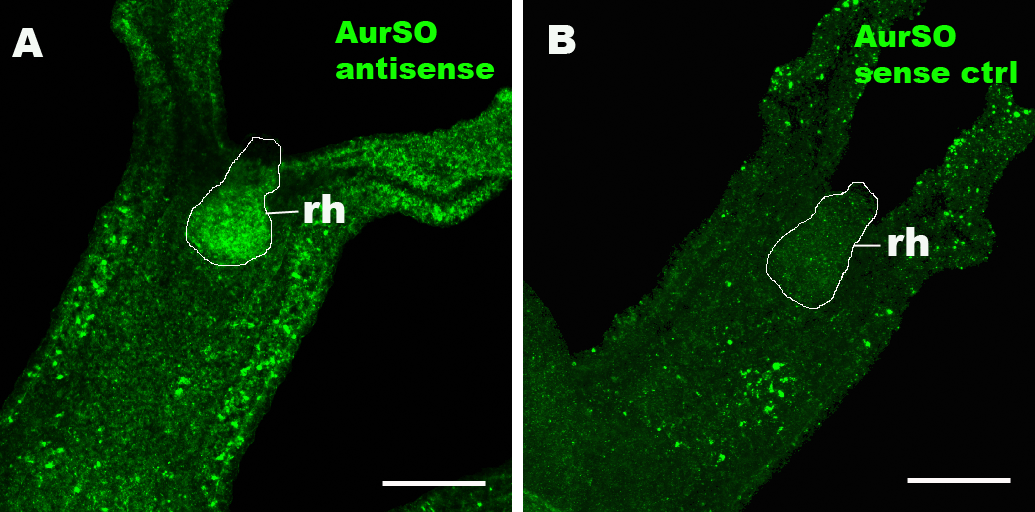

Supplement: S10 Fig — Confocal sections through rhopalia (rh) in Aurelia sp.1 ephyrae fluorescently labeled with an antisense riboprobe against AurSO (A), and with an AurSO sense riboprobe (B) as a control. The specimens are viewed orally, and the distal side is up. The rhopalia are outlined in white. Strong labeling occurs in rhopalia (rh) when an antisense probe is used (A), but not when a sense probe is used (B). Scale bars: 100 μm. (TIF) [file pone.0132544.s010.tif]
